# A National Survey of Kidney Supportive Care Services in Ireland

**DOI:** 10.1016/j.ekir.2026.107072

**Published:** 2026-09-08

**Authors:** Sorcha O’Brien, Jacob Mackey, Barnaby Hole, Kathryn Ducharlet, Brendan Smyth, John Curtin, Eva B. Long, Sarah M. Moran, Aoife C. Lowney

**Affiliations:** 1 Department of Renal Medicine, Cork University Hospital, Ireland; 2 School of Medicine, University College Cork, Cork, Ireland; 3 Population Health Sciences, University of Bristol, UK; 4 UK Renal Registry, Southmead Hospital, Bristol, UK; 5 Department of Renal Medicine, Eastern Health, Victoria, Australia; 6 Eastern Health Clinical School, Monash University, Victoria, Australia; 7 Department of Renal Medicine, St George Hospital, Australia; 8 Department of Palliative Care, Cork University Hospital, Ireland; 9 Marymount University Hospital and Hospice, Cork, Ireland

**Keywords:** dialysis, kidney supportive care, palliative care, patient-centered care

## Introduction

Kidney supportive care (KSC) integrates nephrology and palliative care principles to optimize symptom control, shared decision-making, advance care planning, and psychosocial support for people with advanced chronic kidney disease (CKD) and kidney failure.^1^, ^2^, ^3^ International guidance recommends embedding KSC within nephrology services, yet the model, staffing, and funding for KSC services vary internationally.^1^^,^^4^ Multinational data from Australia, Aotearoa-New Zealand, and the United Kingdom described wide variation in models of KSC and heterogeneity in staffing and activities despite common clinical priorities.^5^ Ireland does not have a national KSC program; understanding how KSC is delivered across Irish kidney units is an essential first step toward informing service development, workforce planning and benchmarking against international practice. We conducted a cross-sectional survey of adult kidney units across Ireland to: (i) characterize models of KSC delivery in Irish units; (ii) describe first-line symptom-management strategies used locally; and (iii) compare Irish practice with contemporary international data to identify gaps for service development. Detailed procedure is given in Supplementary Methods.

## Results

Sixteen adult kidney units were invited to participate, and 14 responded to the survey (88% response rate). Table 1 summarizes unit participation.

**Table 1:** Unit responses to survey

Unit response	*n* (%)
No. of all kidney units	16 (100)
No. of contacted units	16 (100)
No. of responding units	14 (88)
No. of units responding to symptom management	14 (88)
No. of units responding to service provision	9 (56)
Units with MDT between nephrology/palliative care team	2 (22)
Units with dedicated KSC team	1 (11)

KSC, kidney supportive care; MDT, multidisciplinary team.

There is 1 dedicated KSC service in Ireland, delivered by a nephrologist contributing 0.25 full-time equivalent and a full-time KSC clinical nurse specialist. Referrals are accepted from nephrology and palliative care physicians and nurse specialists, with no specific referral criteria. The service receives an estimated 3 new referrals and reviews 12 patients monthly. The cohort is predominantly older and male, with 84% of patients aged 70 or older (56% aged 80 or older) and 64% male. This service is funded entirely by the parent hospital.

Symptom assessment is conducted using the IPOS-Renal questionnaire at initial assessment and when new symptoms arise. Frailty is assessed using the Karnofsky Performance Status and the Rockwood Clinical Frailty Scale.

Across responding units, nephrologists and dialysis nurses were the principal KSC providers (Table 2). Specialist hospital or community palliative care teams were involved in 5 of 9 units (56%). Seven units estimated their conservatively managed population (range, 15–300); 4 units reported discharging a small proportion of conservatively managed patients back to primary care (2%–10%).

**Table 2:** Delivery of KSC in Ireland

Delivery of KSC	*n* (%), *N* = 9
Nephrologists	8 (89)
Unit nursing staff	8 (89)
Specialist palliative care service	5 (56)
Community palliative care service	5 (56)
General practitioners	4 (44)
Dieticians	2 (22)
Social work	2 (22)
Physiotherapy	1 (11)

Symptom-management practices varied across units (Supplementary Figures S1–S8).

Compared with Marsh *et al.*,^5^ Ireland has fewer dedicated KSC services (Ireland: 1 of 16 units in our sample; Marsh *et al.*^5^: 66% of responding units across Australia/Aotearoa New Zealand /United Kingdom had a KSC service). Staffing patterns also differ: Marsh and colleagues reported that KSC services commonly included specialist KSC nurses, dietitians, and social workers in addition to nephrologists,^5^ whereas our Irish data indicate that nephrologists and nursing staff provide the majority of KSC activity, with less frequent allied health and palliative care staffing.

### Therapeutic Management of Pain

For non-neuropathic pain, the most commonly used first-line therapy was buprenorphine (see Supplementary Figure S1). Interestingly, 3 units reported using codeine as a first-line therapy and 1 unit reported using morphine as first-line for non-neuropathic pain management, despite guidelines advising against codeine and morphine use in CKD.

These results vary from the multinational study,^6^ where oxycodone was the most widely used first-line therapy for nonneuropathic pain overall. The discussion in the aforementioned study notes that hydromorphone is available in the United Kingdom but was not used there as it is in Australia. Although international guidelines favor hydromorphone over oxycodone, its low-strength formulation is available in Australia and New Zealand but not in the United Kingdom or Ireland, likely explaining this difference.^7^

### Therapeutic Management of Chronic Kidney Disease Associated Pruritus

For topical therapies, menthol and capsaicin were used most frequently as first-line therapies, with lignocaine and hydrocortisone used the most overall (see Supplementary Figure S5). These results are similar to the international study,^8^ with menthol and capsaicin also being the most frequently used first-line therapies.

As for systemic therapies, diphenhydramine (used in 40% of units) was the most common first-line treatment (see Supplementary Figure S6). However, gabapentin and pregabalin were used the most overall, with all units using them in some capacity. In the United Kingdom, Australia, and New Zealand study,^8^ gabapentinoids were most commonly used first line treatment and overall.

### Descriptive Analysis of Common Themes

A review of the free text answers, using approaches informed by Braun and Clarke thematic analysis,^9^ identified 2 key themes: (1) Education and staffing of a dedicated KSC service and (2) increasing demand for a kidney supportive care service.

### Education and Staffing of a Dedicated KSC Service

Many respondents expressed interest in developing a dedicated kidney supportive care service; however, they highlighted funding and staff education as key barriers.

“If properly supported and resourced, this would undoubtedly improve the care patients receive…”.

“…it would be good to have a dedicated renal palliative care nurse.”

### Increasing Demand for a Dedicated KSC Service

Respondents highlighted increasing demand for a KSC service in the context of an aging, increasingly comorbid population.

“As we have more elderly and patients with multiple comorbidities, it would be helpful to have a dedicated team.”

Respondents highlighted published literature that demonstrates improved care when provided by a dedicated service.

“… and likely improved quality of life and reduce premature or inappropriate dialysis initiation.”

“Literature supports a higher quality of service by a dedicated team.”

## Discussion

This national survey provides the first systematic description of KSC provision across adult kidney units in Ireland. KSC is largely delivered informally by nephrologists and dialysis nurses in most units, with limited availability of dedicated KSC teams or multidisciplinary structures. Practice varies considerably, particularly in the management of CKDaP, highlighting the need for national guidelines. Key barriers identified to developing a dedicated KSC service include funding and staff training, although there is strong interest in developing a collaborative KSC team to improve patient care and quality of life.

Marsh *et al.*^5^ described a more widespread presence of dedicated KSC teams across Australia, New Zealand, and the United Kingdom, and a broader allied-health composition including specialist KSC nurses, dietitians, and social workers. Differences between Ireland and those countries may reflect differences in funding, workforce capacity, healthcare structure, and the maturity of KSC services. Ireland’s reliance on nephrologists and dialysis nurses for KSC may represent a pragmatic use of available skills but also highlights opportunities to expand multidisciplinary input and formalize service structures.

Implications for Irish service development include prioritizing: (i) workforce planning for specialist KSC roles, (ii) formal multidisciplinary clinics or regular nephrology–palliative care multidisciplinary teams, (iii) standardization of symptom-assessment tools and outcome measurement, and (iv) national guidance for symptom management to reduce unwarranted variation. The ISN curriculum and consensus definitions for KSC/CKM,^1^^,^^4^ as well as the Australian and New Zealand guidance documentS1 provide helpful frameworks to support local implementation, which we suggest should be incorporated into the Royal College of Physicians Ireland curriculum for nephrology and palliative care trainees. Future research should evaluate the effect of different KSC models on patient-reported outcomes, symptom burden, healthcare utilization, and cost-effectiveness.

### Strengths and Limitations

Strengths include a high response rate and use of a survey instrument previously used internationally, allowing direct comparison. Limitations include the sample size and potential response bias: units with an interest in KSC may have been more likely to participate. Responses from clinical directors may not reflect practice across all nephrologists, although the survey was allowed to be shared among unit staff. The reported number of conservatively managed patients varied widely (15–300), potentially reflecting differing definitions, as no definition was provided. Partially completed responses limited quantitative comparisons for some therapies. Finally, the Marsh *et al.*^5^ dataset was collected earlier and from different health systems; therefore comparisons should be interpreted cautiously.

## Conclusion

KSC in Ireland is predominantly delivered by nephrologists and dialysis nurses, with variable palliative-care and allied-health involvement. Strong interest exists in developing dedicated KSC services. The findings provide a national baseline to inform workforce planning, service design, and international benchmarking. Standardized assessment, data collection, and piloting of dedicated KSC roles are recommended next steps.

## Disclosures

BS received honoraria for speaking and participated on an advisory board for CSL Seqirus.

## Supplementary Material

Supplementary File (PDF) **Supplementary Methods. Supplementary Reference. Figure S1.** Therapies used for non-neuropathic moderate to severe pain (*n* = 13). **Figure S2.** Therapies used for neuropathic moderate to severe pain (*n* = 13). **Figure S3.** Therapies used for insomnia (*n* = 14). **Figure S4.** Therapies used for nausea and vomiting (*n* = 13). **Figure S5.** Topical therapies used for itch (*n* = 12). **Figure S6.** Systemic therapies used for itch (*n* = 12). **Figure S7.** Therapies used for restless leg syndrome (*n* = 12). **Figure S8.** Therapies used for muscle cramps (*n* = 14). Copy of REDCap survey of kidney supportive practices. CROSS Checklist.

## Footnotes

Supplementary File (PDF)
**Supplementary Methods**. **Supplementary Reference**. **Figure S1.** Therapies used for non-neuropathic moderate to severe pain (*n* = 13). **Figure S2.** Therapies used for neuropathic moderate to severe pain (*n* = 13). **Figure S3.** Therapies used for insomnia (*n* = 14). **Figure S4.** Therapies used for nausea and vomiting (*n* = 13). **Figure S5.** Topical therapies used for itch (*n* = 12). **Figure S6.** Systemic therapies used for itch (*n* = 12). **Figure S7.** Therapies used for restless leg syndrome (*n* = 12). **Figure S8.** Therapies used for muscle cramps (*n* = 14). Copy of REDCap survey of kidney supportive practices. CROSS Checklist.
