# Supplementary material for: A National Survey of Kidney Supportive Care Services in Ireland
Source: Kidney Int Rep. 2026 Sep 8;11(11):107072. doi: 10.1016/j.ekir.2026.107072 (PMC13672975; doi:10.1016/j.ekir.2026.107072)
Supplement: Supplementary file 1 [file mmc1.pdf]

Supplementary Methods

We conducted an observational cross-sectional survey of adult dialysis units in Ireland, using a survey adapted from a KSC survey conducted in Aotearoa-Australia, New Zealand and the United Kingdom.[5] All Irish units providing hemodialysis to adults with end-stage kidney disease were identified through the National Renal Office website, and clinical directors were invited via email to participate. The survey could be shared with relevant unit staff and associated satellite dialysis units to be completed by the most appropriate person or persons.

Supplementary Methods

We conducted an observational cross-sectional survey of adult kidney/dialysis units in Ireland. Ethical approval was obtained from the Clinical Research Ethics Committee, University College Cork (CREC). The survey instrument was adopted, with permission, from a KSC survey published from Australia, New Zealand, and the UK [5]; it had been assessed for readability, feasibility and face validity in four international KSC services and further reviewed locally by one Irish nephrologist and one palliative care consultant prior to deployment, with no significant changes required to the survey. Units were identified from the National Renal Office website, and clinical directors of the dialysis units were invited via email to participate. The survey was distributed using REDCap. Links could be shared among unit staff to permit the various parts of the survey to be completed by the most appropriate person or people within the unit. Recruitment and data collection occurred between December 2024 and January 2025. Individual round two emails were sent to the directors of units that did not respond after a one-month period, which resulted in two further responses. A third attempt at contact was made to the remaining two units, which did not result in response after a further two weeks, at which point the survey was closed. The survey captured five domains: symptom management, availability and organisation of KSC, roles of KSC services, KSC activity, and use of standardised assessments. Questions employed categorical choices, Likert scales and free-text fields. All returned surveys, including those partially completed, were included in the analysis; for partially answered Likert questions, remaining selections were treated as ‘never used’ for descriptive reporting. Free-text responses were interpreted qualitatively and common themes identified were described. Descriptive statistics (frequencies and proportions) were calculated in Microsoft Excel. Where appropriate, we compared Irish findings with the multinational dataset reported previously [5-7]; statistical comparison was not attempted given differences in sampling frames and survey timing.

Supplementary Figures

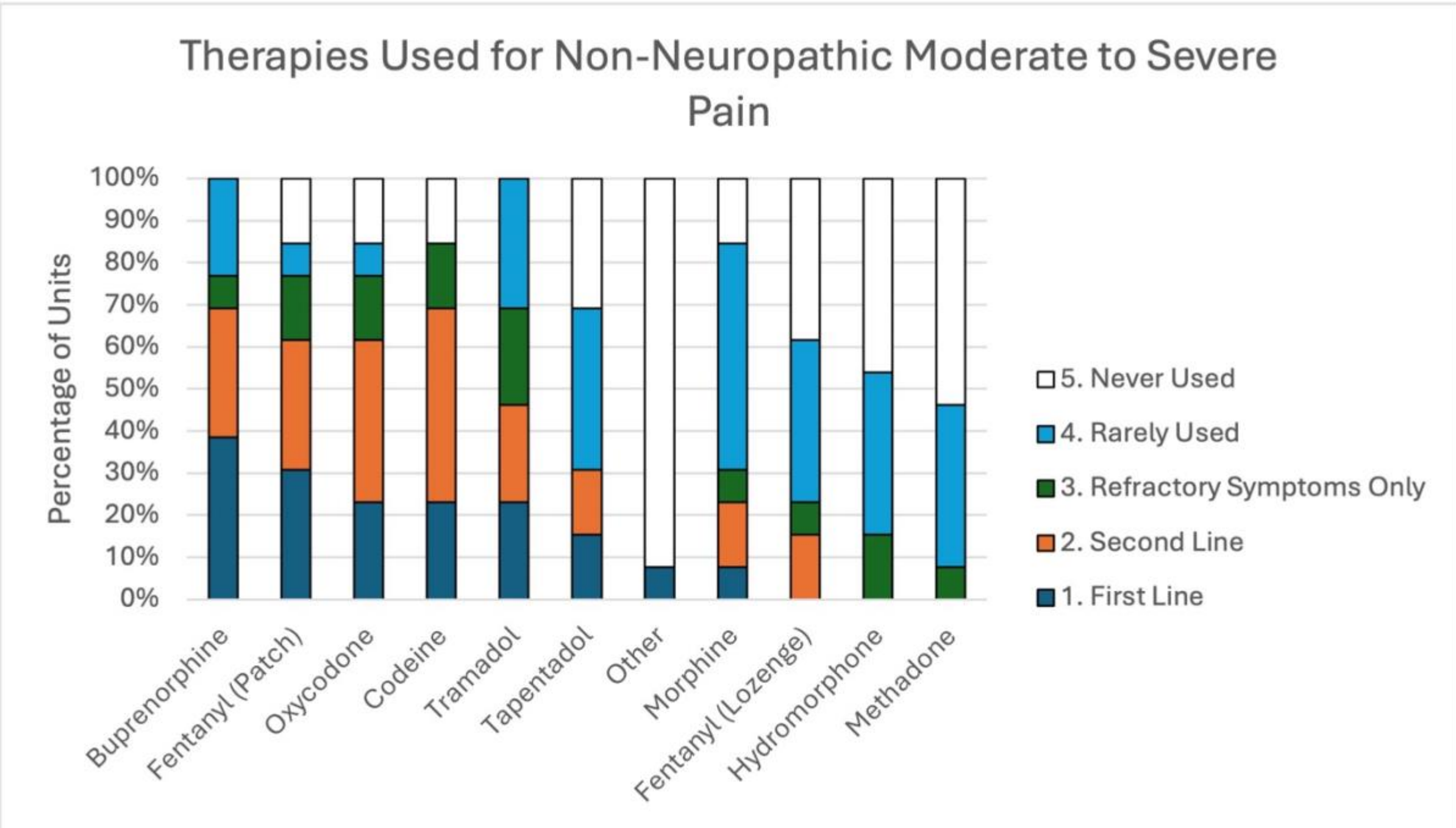

Supplementary Figure S1: Therapies used for non-neuropathic moderate to severe pain (n=13).

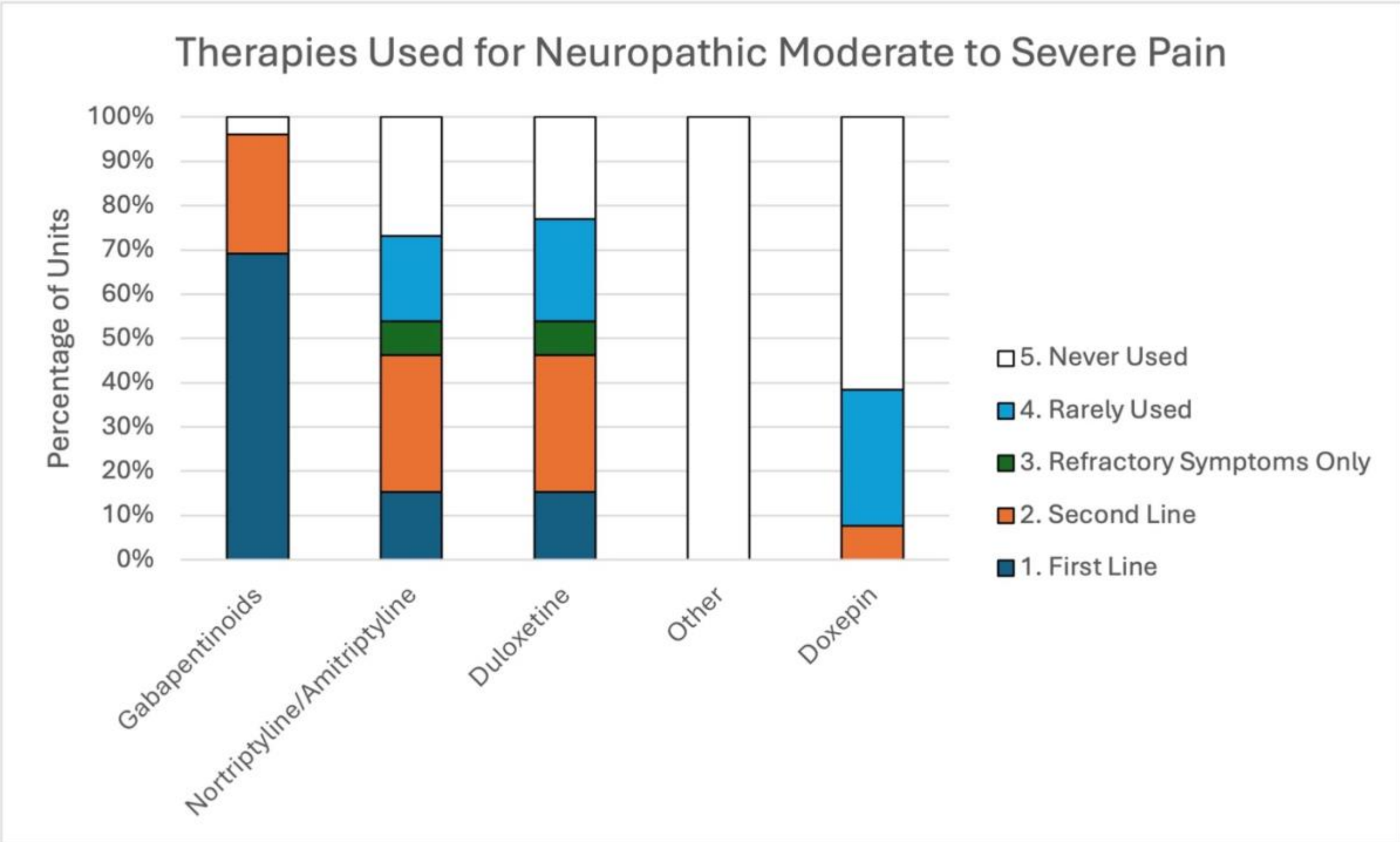

Supplementary Figure S2: Therapies used for neuropathic moderate to severe pain (n=13).

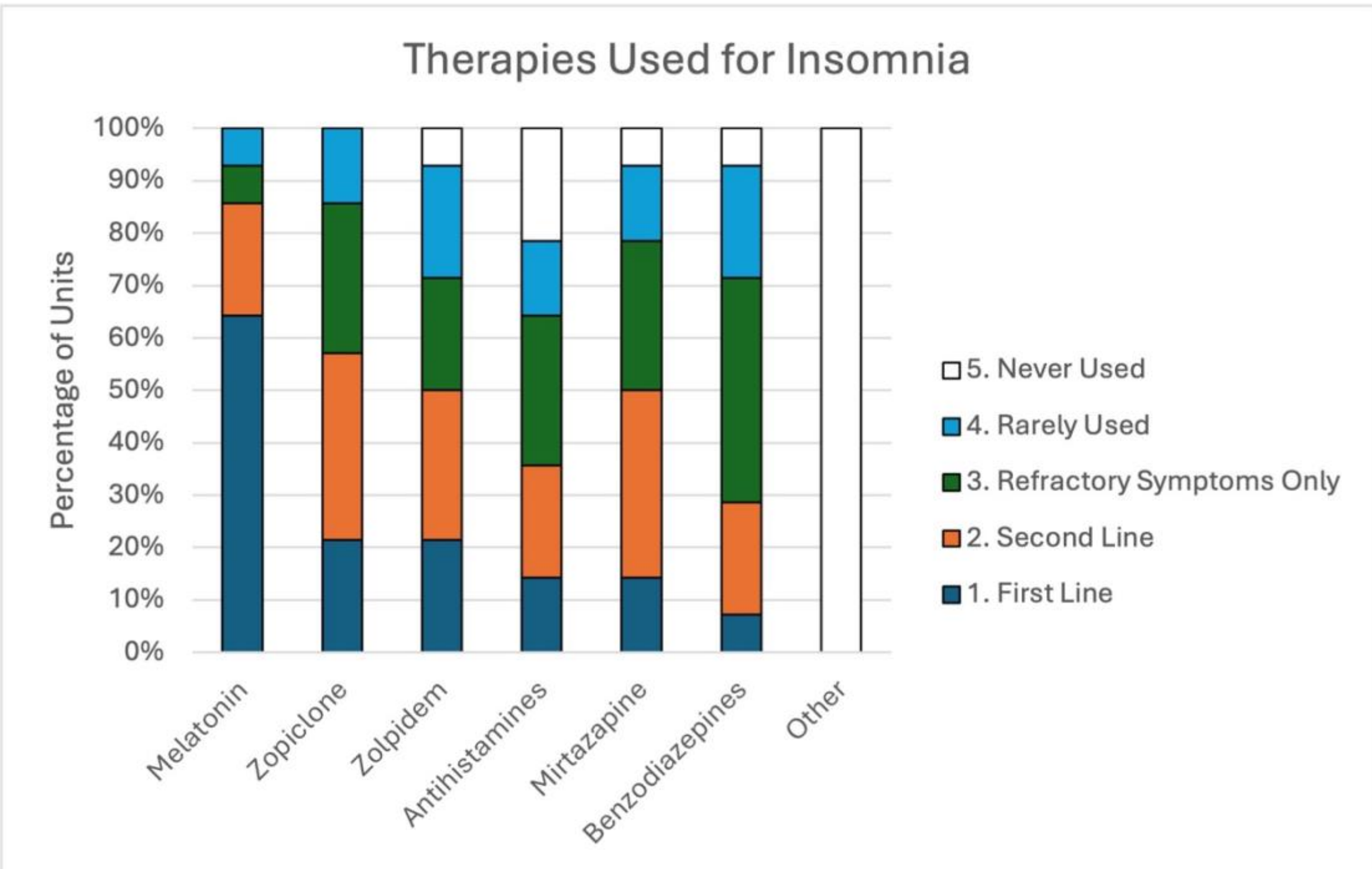

Supplementary Figure S3: Therapies used for insomnia (n=14).

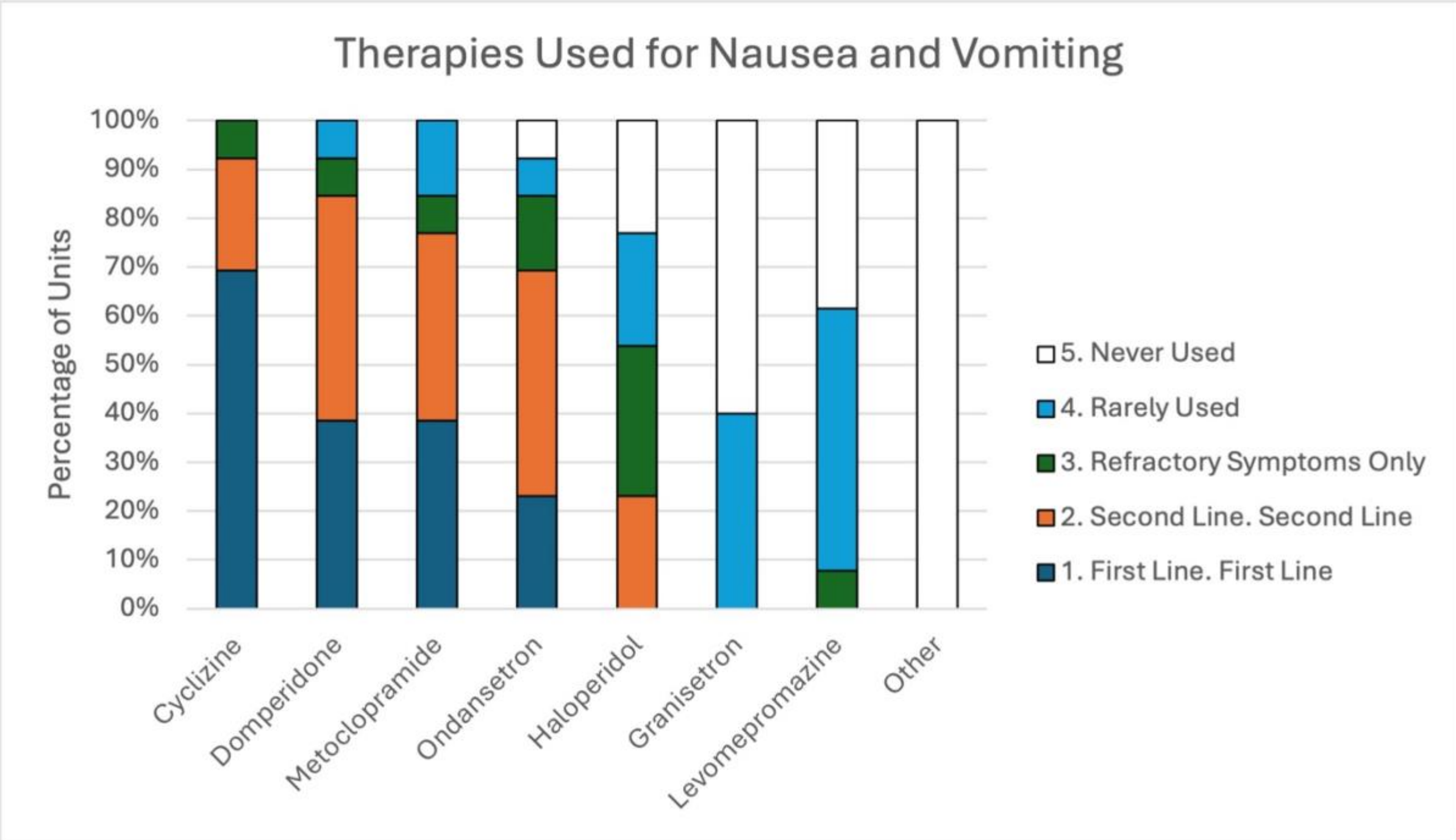

Supplementary Figure S4: Therapies used for nausea and vomiting (n=13).

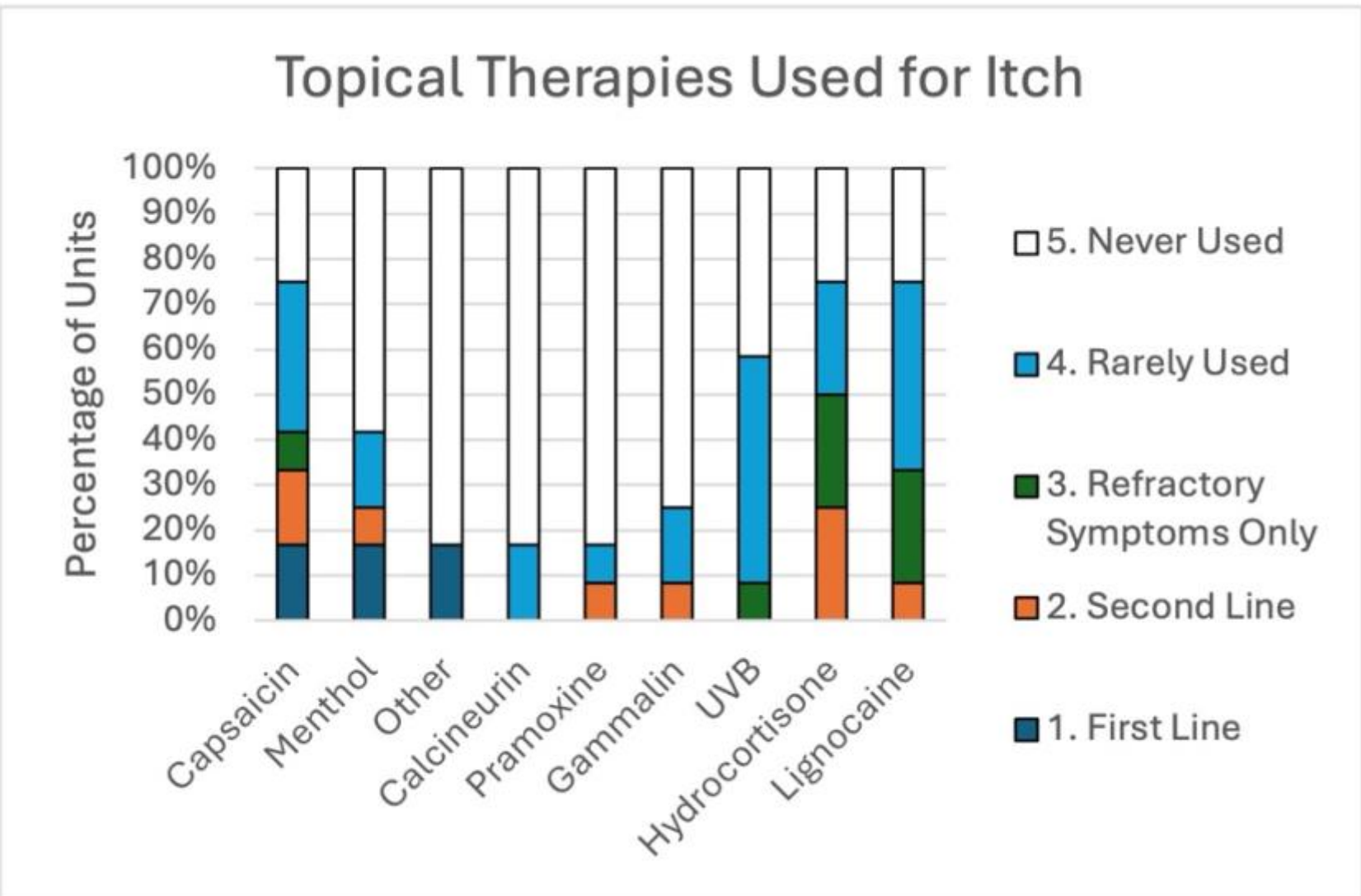

Supplementary Figure S5: Topical therapies used for itch (n=12).

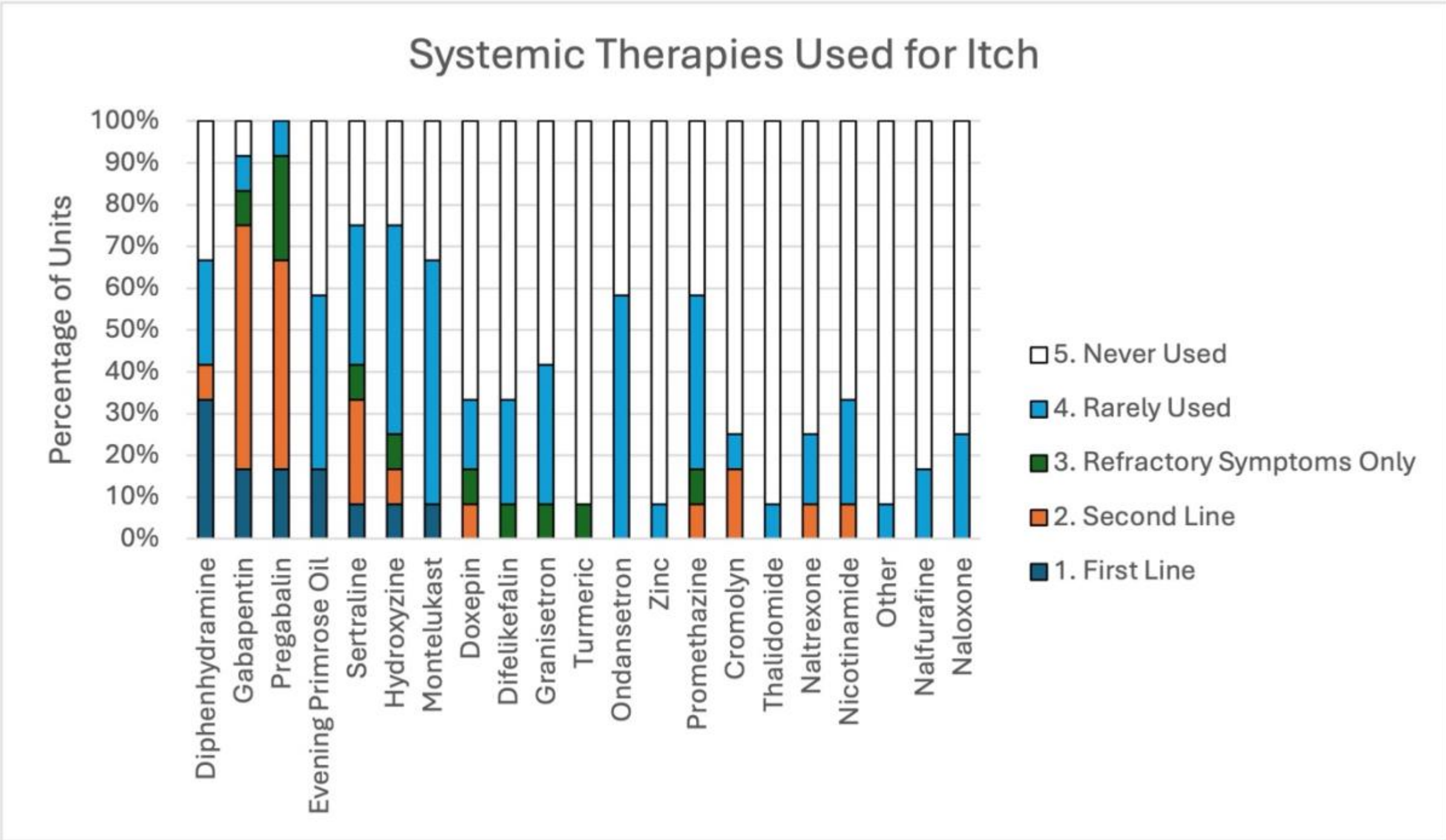

Supplementary Figure S6: Systemic therapies used for itch (n=12).

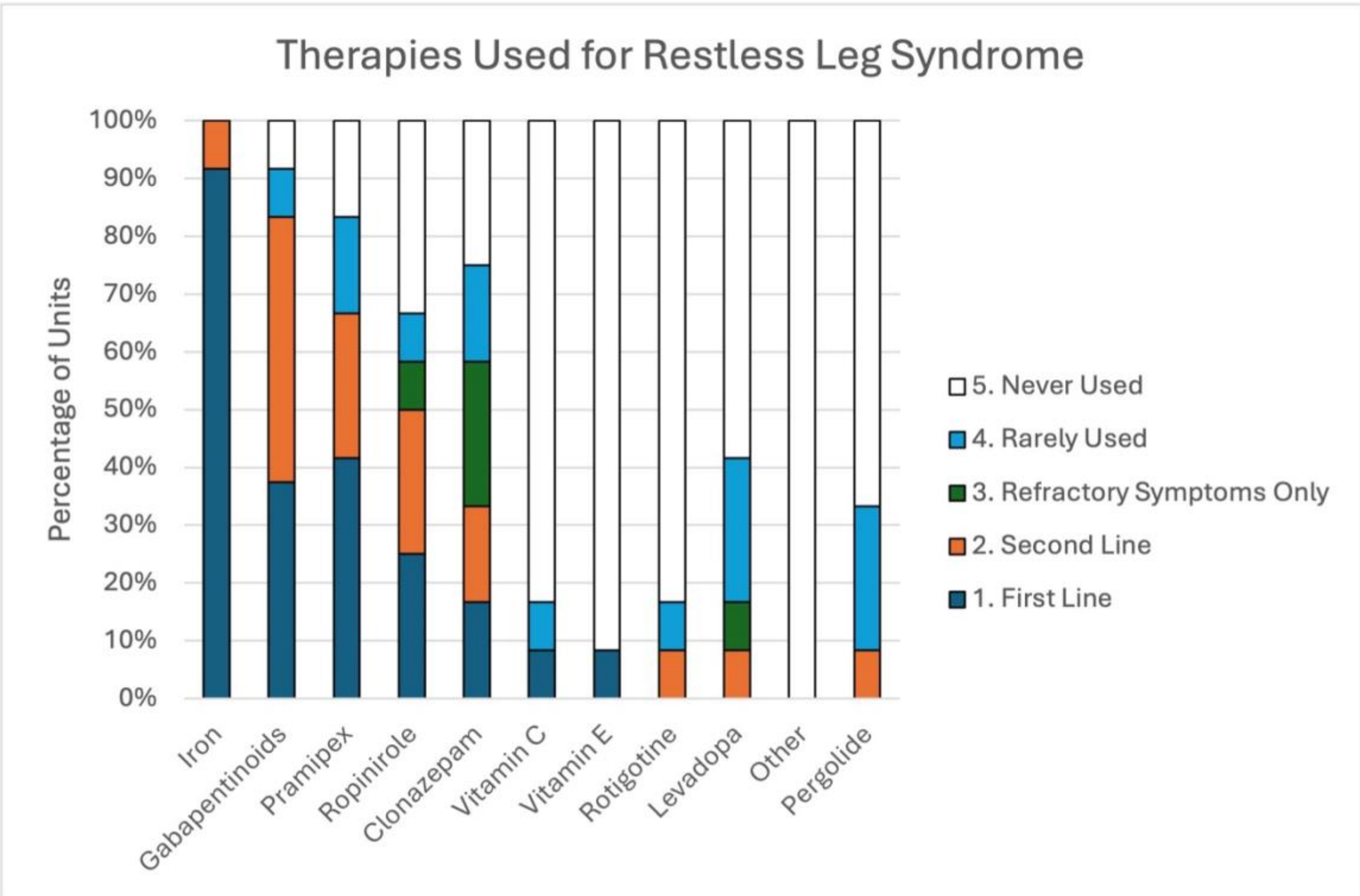

Supplementary Figure S7: Therapies used for restless leg syndrome (n=12).

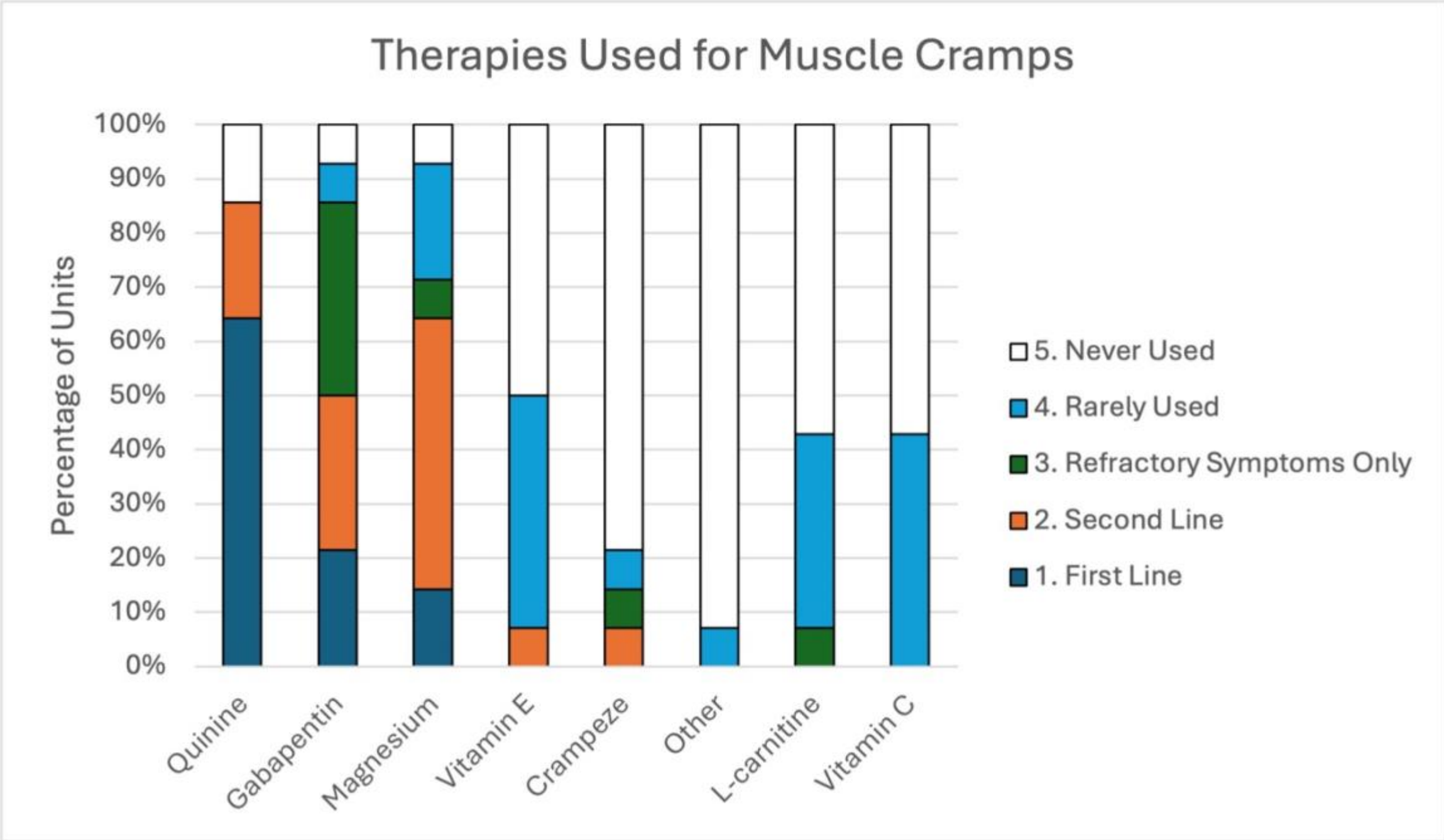

Supplementary Figure S8: Therapies used for muscle cramps (n=14).

Introduction

Thank you in advance for completing this survey of Renal Supportive Care treatment practices, activity, and resourcing.

Multiple staff members at your unit can contribute to completing this survey. So please feel free to share the link provided to you with those who are best placed to answer each section.

Please refer to the Participant Information Sheet (below) for an explanation of how the data you provide will be used and protected.

At any time you or others can save the survey and return to complete it later.

The sections are as follows:
Section A: Symptom Management
Section B: Availability and Organisation of Kidney/Renal Supportive Care (RSC) Resources
Section C: The Role of the RSC Service
Section D: RSC Service Activity Section E: Standardised Assessments

Sections A and B will be relevant to all units and should be completed first, which will require approximately 15 minutes. Units with a dedicated Renal Supportive Care Service will then be asked to go on to complete Sections C, D and E, which will require an additional 30 to 45 minutes.

Thank you!

Dr Neeru Agarwal, NHMRC Clinical Trial Centre

Dr Kathryn Ducharlet, Monash University

Dr Daniel O'Hara, NHMRC Clinical Trial Centre

Dr Brendan Smyth, NHMRC Clinical Trial Centre

Please enter the name of your unit

____

Section A: Symptom Management

Section A: Symptom Management

These questions relate to the management of symptoms in patients with advanced chronic kidney disease or kidney failure. This includes patients receiving kidney replacement therapy (KRT), patients not yet receiving KRT but planning for it, and patients planning for or receiving comprehensive conservative care.

Unless otherwise stated, these questions relate to typical practice at your unit. While we understand that you may not know exactly what each of your unit colleagues would do in the scenarios provided, we ask that you answer with what is, to the best of your knowledge, usual practice at your unit.

Please feel free to comment on variation in practice between clinicians in your unit for management of a given symptom in the free text boxes provided.

A1. For patients with altered taste, which of the following treatments are in use at your unit?
(You can select multiple agents for each category, for example, multiple agents can be 'second line' if that best represents your practice. This question assumes that changing the flavour profile of food has been recommended.)

	First line	Second line	Refractory symptoms only	Rarely used	Never used
Chewing gum	○	○	○	○	○
Menthol products	○	○	○	○	○
Sodium bicarbonate mouthwash	○	○	○	○	○
Zinc supplementation	○	○	○	○	○
Other (specify) ______	○	○	○	○	○
Other (specify) ______	○	○	○	○	○

Do you have any additional comments?

_____ _____

A2. For patients with skeletal muscle cramps, which of the following treatments are in use at your unit?

(You can select multiple agents for each category, for example, multiple agents can be 'second line' if that best represents your practice. This question assumes that all

non-pharmacological measures such as exercise, stretching, and minimising intra-dialytic weight gain have been recommended.)

	First line	Second line	Refractory symptoms only	Rarely used	Never used
Crampeze (Magnesium/Viburnum Opulus/Ginkgo Biloba)	◯	◯	◯	◯	◯
Gabapentin	◯	◯	◯	◯	◯
L-Carnitine	◯	◯	◯	◯	◯
Magnesium	◯	◯	◯	◯	◯
Quinine	◯	◯	◯	◯	◯
Vitamin C	◯	◯	◯	◯	◯
Vitamin E	◯	◯	◯	◯	◯
Other (specify) ______	◯	◯	◯	◯	◯
Other (specify) ______	◯	◯	◯	◯	◯

Do you have any additional comments?

_____ _____

A3. For patients with fatigue, which of the following treatments are in use at your unit? (You can select multiple agents for each category, for example, multiple agents can be 'second line' if that best represents your practice. This question assumes that correction of anaemia and iron deficiency have been addressed, exercise and sleep hygiene practices have been recommended, and that symptoms that disturb sleep such as Restless Legs Syndrome, pain and pruritis have been addressed.)

	First line	Second line	Refractory symptoms only	Rarely used	Never used
Human Growth Hormone (e.g. norditropin)	◯	◯	◯	◯	◯
L-Carnitine	◯	◯	◯	◯	◯
Nandrolone decanoate (anabolic steroid)	◯	◯	◯	◯	◯
Other (specify) ______	◯	◯	◯	◯	◯
Other (specify) ______	◯	◯	◯	◯	◯

Do you have any additional comments?

_____ _____

A4. For patients with insomnia, which of the following treatments are in use at your unit? (You can select multiple agents for each category, for example, multiple agents can be 'second line' if that best represents your practice. This question assumes that

non-pharmacological measures have been recommended.)

	First line	Second line	Refractory symptoms only	Rarely used	Never used
Benzodiazepines	○	○	○	○	○
Melatonin	○	○	○	○	○
Mirtazepine	○	○	○	○	○
Sedating antihistamine (e.g. promethazine)	○	○	○	○	○
Zolpidem	○	○	○	○	○
Zopiclone	○	○	○	○	○
Other (specify) ______	○	○	○	○	○
Other (specify) ______	○	○	○	○	○

Do you have any additional comments?

_____ _____

A5. For patients with nausea or vomiting, which of the following treatments are in use at your unit?

(You can select multiple agents for each category, for example, multiple agents can be 'second line' if that best represents your practice. This question assumes that

non-pharmacological measures such as eating smaller and more frequent meals, and

addressing constipation, have been explored.)

	First line	Second line	Refractory symptoms only	Rarely used	Never used
Cyclizine	○	○	○	○	○
Domperidone	○	○	○	○	○
Granisetron	○	○	○	○	○
Haloperidol	○	○	○	○	○
Levomepromazine	○	○	○	○	○
Metoclopramide	○	○	○	○	○
Ondansetron	○	○	○	○	○
Other (specify) ______	○	○	○	○	○
Other (specify) ______	○	○	○	○	○

Do you have any additional comments?

_____ _____

A6. For patients with moderate to severe chronic pain, which of the following treatments are in use at your unit?

Non-neuropathic and neuropathic pain are considered separately.

(You can select multiple agents for each category, for example, multiple agents can be 'second line' if that best represents your practice. This question assumes that

non-pharmacological measures, or specific treatments to address the source of the pain, have

been explored. This question is not intended to cover end-of-life pain management where practice may differ)

Non-Neuropathic Pain

	First line	Second line	Refractory symptoms only	Rarely used	Never used
Buprenorphine (transdermal)	○	○	○	○	○
Codeine	○	○	○	○	○
Fentanyl (lozenge)	○	○	○	○	○
Fentanyl (transdermal)	○	○	○	○	○
Hydromorphone	○	○	○	○	○
Methadone	○	○	○	○	○
Morphine	○	○	○	○	○
Oxycodone	○	○	○	○	○
Tapentadol	○	○	○	○	○
Tramadol	○	○	○	○	○
Other (specify) ______	○	○	○	○	○
Other (specify) ______	○	○	○	○	○

Neuropathic Pain

	First line	Second line	Refractory symptoms only	Rarely used	Never used
Amitryptiline	○	○	○	○	○
Doxepin	○	○	○	○	○
Duloxetine	○	○	○	○	○
Gabapentin	○	○	○	○	○
Nortryptiline	○	○	○	○	○
Pregabalin	○	○	○	○	○
Other (specify) ______	○	○	○	○	○
Other (specify) ______	○	○	○	○	○

Do you have any additional comments?

______ ______

A7. For patients with pruritus, which of the following systemic and topical treatments are in use at your unit?

(You can select multiple agents for each category, for example, multiple agents can be 'second line' if that best represents your practice. This question assumes that

non-pharmacological measures such as simple skin care and emollients have already been

recommended.)

Systemic Treatments for Pruritus

	First line	Second line	Refractory symptoms only	Rarely used	Never used
Cromolyn	○	○	○	○	○
Difelikefalin	○	○	○	○	○
Diphenhydramine	○	○	○	○	○
Doxepin	○	○	○	○	○
Evening Primrose Oil	○	○	○	○	○
Gabapentin	○	○	○	○	○
Granisetron	○	○	○	○	○
Hydroxyzine	○	○	○	○	○
Montelukast	○	○	○	○	○
Nalfurafine	○	○	○	○	○
Naloxone	○	○	○	○	○
Naltrexone	○	○	○	○	○
Nicotinamide	○	○	○	○	○
Ondansetron	○	○	○	○	○
Pregabalin	○	○	○	○	○
Promethazine	○	○	○	○	○
Sertraline	○	○	○	○	○
Thalidomide	○	○	○	○	○
Turmeric	○	○	○	○	○
Zinc	○	○	○	○	○
Other (specify) ______	○	○	○	○	○
Other (specify) ______	○	○	○	○	○

Topical Treatments for Pruritus

	First line	Second line	Refractory symptoms only	Rarely used	Never used
Capsaicin	○	○	○	○	○
Calcineurin inhibitor	○	○	○	○	○
Gamma-linolenic acid	○	○	○	○	○
Hydrocortisone	○	○	○	○	○

Lignocaine/lidocaine	◯	◯	◯	◯	◯
Menthol	◯	◯	◯	◯	◯
Pramoxine	◯	◯	◯	◯	◯
UV B light therapy	◯	◯	◯	◯	◯
Other (specify) ______	◯	◯	◯	◯	◯
Other (specify) ______	◯	◯	◯	◯	◯

Do you have any additional comments?

_____ _____

A8. For patients with restless legs syndrome, which of the following treatments are in use at your unit?

(You can select multiple agents for each category, for example, multiple agents can be 'second line' if that best represents your practice. This question assumes that

non-pharmacological measures have been recommended.)

	First line	Second line	Refractory symptoms only	Rarely used	Never used
Clonazepam	◯	◯	◯	◯	◯
Gabapentin	◯	◯	◯	◯	◯
Iron replacement (if not iron replete)	◯	◯	◯	◯	◯
Levodopa / Carbidopa	◯	◯	◯	◯	◯
Pergolide	◯	◯	◯	◯	◯
Pramipexole	◯	◯	◯	◯	◯
Pregabalin	◯	◯	◯	◯	◯
Ropinirole	◯	◯	◯	◯	◯
Rotigotine	◯	◯	◯	◯	◯
Vitamin C	◯	◯	◯	◯	◯
Vitamin E	◯	◯	◯	◯	◯
Other (specify)	◯	◯	◯	◯	◯
Other (specify)	◯	◯	◯	◯	◯

Do you have any additional comments?

_____ _____

A9. Role of Medicinal Cannabis / Cannabinoid Products

In your unit, is medicinal cannabis / cannabinoid products prescribed to people with chronic kidney disease?

◯ Yes - often
◯ Yes - rarely
◯ No
◯ I don't know

What symptoms are medicinal cannabis/cannabinoid products prescribed for? Select all that apply

☐ Pruritus
☐ Neuropathic pain
☐ Non-neuropathic pain
☐ Nausea and vomiting
☐ Restless leg syndrome
☐ Insomnia
☐ Anorexia
☐ Fatigue
☐ Anxiety
☐ Other (specify)

Please specify other symptom managed with medicinal cannabis / cannabinoid products

____

	First line symptoms	Second line	Refractory only	Rarely used
What role does medicinal cannabis / cannabinoid products play in PRURITUS management in your unit?	◯	◯	◯	◯

	First line symptoms	Second line	Refractory only	Rarely used
What role does medicinal cannabis / cannabinoid products play in NEUROPATHIC PAIN management in your unit?	◯	◯	◯	◯

	First line symptoms	Second line	Refractory only	Rarely used
What role does medicinal cannabis / cannabinoid products play in NON-NEUROPATHIC PAIN management in your unit?	◯	◯	◯	◯

	First line symptoms	Second line	Refractory only	Rarely used
What role does medicinal cannabis / cannabinoid products play in NAUSEA AND VOMITING management in your unit?	◯	◯	◯	◯

What role does medicinal cannabis / cannabinoid products play in RESTLESS LEGS management in your unit?	First line symptoms ◯	Second line ◯	Refractory only ◯	Rarely used ◯
What role does medicinal cannabis / cannabinoid products play in INSOMNIA management in your unit?	First line symptoms ◯	Second line ◯	Refractory only ◯	Rarely used ◯
What role does medicinal cannabis / cannabinoid products play in ANOREXIA management in your unit?	First line symptoms ◯	Second line ◯	Refractory only ◯	Rarely used ◯
What role does medicinal cannabis / cannabinoid products play in FATIGUE management in your unit?	First line symptoms ◯	Second line ◯	Refractory only ◯	Rarely used ◯
What role does medicinal cannabis / cannabinoid products play in ANXIETY management in your unit?	First line symptoms ◯	Second line ◯	Refractory only ◯	Rarely used ◯
What role does medicinal cannabis / cannabinoid products play in the management of [cannabisothersymptom] in your unit?	First line symptoms ◯	Second line ◯	Refractory only ◯	Rarely used ◯

Would your unit have any interest in participating in a clinical trial of medicinal cannabis / cannabinoid products for symptom management in Renal Supportive Care?

◯ Yes ◯ No ◯ I don't know

Do you have any additional comments?

____

To mark this section as complete and return to the list of Sections, please click "Submit"

If you have not completed this section, please click "Save and Return Later" to save your answers, then when you return to the survey you can click "Survey Queue" in the top right corner

Section B: Availability and Organisation of Kidney/Renal Supportive Care (RSC) Resources

SECTION B: Availability and Organisation of Kidney/Renal Supportive Care (RSC) Resources

Please consider all aspects of renal supportive (including palliative) care when answering these questions, including symptom management, quality of life, physical function, shared decision making and advance care planning, as well as spiritual, psychological, family and social support.

As with section A, all questions relate to the care of patients with advanced chronic kidney disease or kidney failure. This includes patients receiving kidney replacement therapy (KRT), patients not yet receiving KRT but planning for it, and patients planning for or receiving comprehensive conservative care.

B1. How many conservatively managed patients with end-stage kidney disease (with eGFR less than 15) does your unit (including all nephrologists) currently manage?

This question refers to your unit as a whole, including a Renal Supportive Care service if you have one. A rough estimate/best guess is acceptable if this information is not easily available.

_____

Number of patients

Does your answer derive from unit records, or is it an estimate, or are you unable to answer? _______

B2. Does your unit have a dedicated RSC team, whose job it is to provide renal supportive care?
We define a renal supportive care team as a team dedicated to the management of symptoms and quality of life issues among those with advanced kidney disease.

- ◯ Yes
- ◯ No

B3. How is renal supportive care provided to patients in your local area?

Select all that apply

- ☐ By unit nephrologists as part of general clinical work
- ☐ By unit nursing staff as part of general clinical work
- ☐ By other staff as part of general clinical work (please specify who) ______
- ☐ Through a specialist palliative care service (hospital based, eg clinics)
- ☐ Through a community palliative care service
- ☐ By general practitioners
- ☐ Other (specify) ______

B4. Approximately what proportion of conservatively managed patients with end-stage kidney disease (with eGFR less than 15) are discharged from your renal service to the care of their general practitioner?

_____%

Proportion of patients

Does your answer derive from unit records, or is it an estimate, or are you unable to answer?

_____

B5. Would you be interested in developing a dedicated renal supportive care service at your unit?

◯ Yes
◯ No

Please comment on your reasons for this choice.

To mark this section as complete and return to the list of Sections, please click "Submit"

If you have not completed this section, please click "Save and Return Later" to save your answers, then when you return to the survey you can click "Survey Queue" in the top right corner

Section C: The Role of the RSC Service

Section C: The Role of the RSC Service

The questions in this section are designed to be completed by units with a designated RSC service.

For each of the following activities, indicate the extent to which they are considered an important part of your RSC service using the following definitions:

For each of the following activities, indicate the extent to which they are considered an important part of your RSC service using the following definitions:

Primary responsibility This is a core activity. RSC often leads this activity.
Secondary responsibility The RSC routinely contributes to this activity, with primary responsibility typically remaining with other healthcare providers, carers, or support services.
Sometimes a responsibility This activity usually happens without the involvement of the RSC service, but RSC is sometimes involved.
Rarely a responsibility This is not a usual activity of the RSC service, but it may be undertaken in exceptional or rare circumstances.
Never a responsibility The RSC does not perform this activity.

	Primary	Secondary	Sometimes	Rarely	Never
122) Symptom management	◯	◯	◯	◯	◯
123) End of life care	◯	◯	◯	◯	◯
124) Care coordination with general practitioners	◯	◯	◯	◯	◯
125) Care coordination with other community services	◯	◯	◯	◯	◯
126) Advance care planning	◯	◯	◯	◯	◯
127) Holding family meetings	◯	◯	◯	◯	◯
128) Discussions about dialysis withdrawal	◯	◯	◯	◯	◯
129) Introduction to palliative care	◯	◯	◯	◯	◯
130) Introduction to renal supportive care	◯	◯	◯	◯	◯
131) Psychological support and care	◯	◯	◯	◯	◯
132) Addressing spiritual and/or cultural aspects of care	◯	◯	◯	◯	◯
133) Provision of interventions to slow progression of CKD	◯	◯	◯	◯	◯
134) Management of metabolic, haematological, and bone and mineral complications of CKD	◯	◯	◯	◯	◯

135)

Support in decision-making between dialysis and conservative pathways	○	○	○	○	○

136) Please describe any additional roles of your renal supportive care service

____

To mark this section as complete and return to the list of Sections, please click "Submit"

If you have not completed this section, please click "Save and Return Later" to save your answers, then when you return to the survey you can click "Survey Queue" in the top right corner

Section D: RSC Service Activity

SECTION D: RSC Service Activity<br><br>**The questions in this section are designed to be completed by units with a designed RSC service. In this section we ask you to address:**<br><br>**RSC Team Members RSC Patient Encounters New Referrals Discharges Patient Demographics Advance Care Planning Other Activities**	
Renal Supportive Care Team Members	
D1. Which clinical staff are involved in delivery of your RSC service?<br><br>Select all that apply	☐ Dietitian<br>☐ Geriatrician<br>☐ IT/data officer<br>☐ Nephrologist<br>☐ Nephrology trainee<br>☐ Occupational therapist<br>☐ Palliative care nurse<br>☐ Palliative care physician<br>☐ Palliative care trainee<br>☐ Physiotherapist<br>☐ Psychiatrist<br>☐ Psychologist or counsellor<br>☐ Religious representative<br>☐ Renal nurse<br>☐ Research nurse or co-ordinator<br>☐ Specialist renal supportive care nurse<br>☐ Social worker<br>☐ Other medical trainee (specify) ______<br>☐ Other medical consultant (specify) ______<br>☐ Other (specify) ______
The following question(s) relate to the Full Time Equivalent (FTE), or proportion of a full time position, dedicated to the RSC.<br><br>This may be more than 1.0 where multiple people are employed in the same/similar role, e.g. if there are two full-time Renal Supportive Care nurses (i.e. FTE=2.0). If no dedicated FTE then please put '0'.	
How much FTE does the Dietitian have for the RSC service?	______________________
How much FTE does the Geriatrician have for the RSC service?	______________________
How much FTE does the IT/Data officer have for the RSC service?	______________________
How much FTE does the Nephrologist have for the RSC service?	______________________
How much FTE does the Nephrology trainee have for the RSC service?	______________________

How much FTE does the Occupational therapist have for the RSC service?	______________________
How much FTE does the Palliative care nurse have for the RSC service?	______________________
How much FTE does the Palliative care physician have for the RSC service?	______________________
How much FTE does the Palliative care trainee have for the RSC service?	______________________
How much FTE does the Physiotherapist have for the RSC service?	______________________
How much FTE does the Psychiatrist have for the RSC service?	______________________
How much FTE does the Psychologist or counsellor have for the RSC service?	______________________
How much FTE does the Religious representative have for the RSC service?	______________________
How much FTE does the Renal nurse have for the RSC service?	______________________
How much FTE does the Research nurse or coordinator have for the RSC service?	______________________
How much FTE does the Specialist renal supportive care nurse have for the RSC service?	______________________
How much FTE does the Social worker have for the RSC service?	______________________
How much FTE does the Other medical trainee have for the RSC service?	______________________
How much FTE does the Other medical consultant have for the RSC service?	______________________
How much FTE does the [d1specify_3] have for the RSC service?	______________________

Renal Supportive Care Patient Encounters

D2. How many different patients are reviewed by your RSC service?

Select the most convenient timeframe for you to report your answer

_____

Number of patients

per

_____

timeframe

Does your answer derive from unit records, or is it an estimate, or are you unable to answer? ______

D3. How many patient encounters are conducted by your RSC service?

Select the most convenient timeframe for you to report your answer

_____

Number of contacts/reviews

per

_____

timeframe

Does your answer derive from unit records, or is it an estimate, or are you unable to answer? ______

Of the [d3nopatients] patient encounters reported per [d3per] in the previous question (D3), how many patients would be seen in each of the following locations / encounter types:

Home visits _____
Inpatient reviews _____
Outpatient clinic appointments _____
Outpatient reviews at the dialysis unit _____
Residential care/nursing home visits

_____
Telephone or video consultation ______
Other ___________

Does your answer derive from unit records, or is it an estimate, or are you unable to answer?_______

D4. Does your RSC service conduct regular outpatient clinics?

- ◯ Yes
- ◯ No

What is the usual frequency of outpatient clinics provided by your RSC service?

Select the most convenient timeframe for you to report your answer

_____

Number of clinics

per

_____

New Referrals

D5. Who can refer a new patient to your RSC clinic?<br>Select as many as apply	☐ General practitioner<br>☐ Nephrologist/trainee<br>☐ Palliative care physician/trainee<br>☐ Other physician<br>☐ Other medical staff<br>☐ Nursing staff<br>☐ Nurse specialists<br>☐ All of the above<br>☐ Other (please specify)______
Do specific referral criteria exist for your RSC service?	◯ Yes<br>◯ No<br>◯ Unsure
Please briefly describe your RSC service referral criteria	____________________________________

D6. How many new patient referrals does your RSC service receive?

Select the most convenient timeframe for you to report your answer

_____

Number of referrals

per

_____

timeframe

Does your answer derive from unit records, or is it an estimate, or are you unable to answer? _______

Discharges from Renal Supportive Care Service

D7. Approximately what proportion of conservatively managed patients with end-stage kidney disease (with eGFR less than 15) are discharged from your RSC service to the care of their general practitioner?

_____%

Proportion of patients

Does your answer derive from unit records, or is it an estimate, or are you unable to answer?

_____

D8. Approximately what proportion of conservatively managed patients with end-stage kidney disease (with eGFR less than 15) are discharged from your RSC service to the care of their usual nephrologist?

_____%

Proportion of patients

Does your answer derive from unit records, or is it an estimate, or are you unable to answer?

_____

Patient Demographics

D9. What proportion (percentage) of patients reviewed by your RSC service fall into each of the following categories of kidney disease?

If convenient, you may select a timeframe to report your answer ______

Does your answer derive from unit records, or is it an estimate, or are you unable to answer? ______

Patients on dialysis ______%
Patients with a kidney transplant _____%
Patients receiving conservative management for kidney failure _____%
Patients with pre-KRT chronic kidney disease ___%
Other __________%
Total (Please click outside of the data entry box to update the total)

_____%

Please check the proportions / percentages in Question D9 add up to 100%

D10. Are any categories of patient not eligible to be seen by your RSC service?

Select all that apply

- ☐ Patients on dialysis
- ☐ Patients with a kidney transplant
- ☐ Patients receiving conservative management for kidney failure
- ☐ Patients with pre-KRT chronic kidney disease
- ☐ Other ineligible group ______
- ☐ All of the above groups are eligible for our RSC service

D11. What proportion (percentage) of patients reviewed by your RSC service fall into each of the following demographic categories?

Please answer as best as you are able. If you are unable to answer all or part, then leave the relevant field(s) blank and move on to the next question.

If convenient, you may select a timeframe to report your answer ______

Age
80 years or older ____%
70-79 years _____%
60-69 years ______%
Younger than 60 years _____%
Gender
Male _____%
Female ______%
Transgender _____%
Other ____%
Indigenous status
Indigenous Australian (Aboriginal or Torres Strait Islander) _____%
Non-indigenous Australian _____%
Please describe where your answers are derived from unit records or are estimates ______

Advance Care Planning

D12. Within each category of patient (as defined below) reviewed by your RSC service, what proportion would have a documented Advanced Care Plan (ACP)?

Please answer as best as you are able, if you are unable to answer all or part, then leave the relevant field(s) blank and move on to the next question.

If convenient, you may select a timeframe to report your answer _______

Patients on dialysis ______%
Patients with a kidney transplant ______%
Patients conservatively management for kidney failure _____%
Patients with pre-KRT chronic kidney disease ___%
Other __________%
Please describe where your answers are derived from unit records or are estimates _______

Other Activities

D13. Does your RSC service conduct regular multidisciplinary meetings (that is, regularly scheduled case management meetings without the patient present)?	◯ Yes<br>◯ No, or not routinely
D14. In the past 12 months, has your RSC service engaged in clinical research?	◯ Yes<br>◯ No
What type(s) of research has your unit been engaged in?<br><br>Select all that apply	☐ Prospective observational<br>☐ Retrospective observational<br>☐ Prospective interventional (includes randomised studies)<br>☐ Qualitative research (e.g. patient interviews, focus groups)<br>☐ Other (specify) ______
D15. How is your RSC service funded?<br><br>Select all that apply. Please leave this question blank if you would prefer not to answer	☐ Funded directly by parent hospital<br>☐ Funded drawn from parent nephrology department<br>☐ Funding drawn from parent palliative care department<br>☐ Funding drawn from both nephrology and palliative care departments<br>☐ Funding from Local Health district<br>☐ Funding from Medicare claims<br>☐ Funding from State health department<br>☐ Other sources of funding (specify)______

To mark this section as complete and return to the list of Sections, please click "Submit"

If you have not completed this section, please click "Save and Return Later" to save your answers, then when you return to the survey you can click "Survey Queue" in the top right corner

Section E: Standardised Assessments

SECTION E: Standardised Assessments	
**This section concerns the routine use of standardised assessments, including patient reported outcome measures (PROMs) such as symptom scores or quality of life scoring tools, and measures of nutrition, function, or frailty, that are used by your RSC service.**	
**E1. Which of the following patient reported outcome measures (PROMs) are routinely used by your RSC service?**	
**Select all that apply**	
Symptom Surveys	☐ IPOS-Renal<br>☐ POS-S Renal<br>☐ ESASr-Renal<br>☐ Other symptoms assessment
Please specify other symptoms assessment	____________________
Quality of Life Surveys	☐ EQ-5D<br>☐ ESRD-SCLTM<br>☐ KDQOL-36<br>☐ KDQOL-SF<br>☐ SF-6D<br>☐ SF-12<br>☐ SF-36<br>☐ Other quality of life survey
Please specify other quality of life survey	____________________
Other Patient Reported Outcome Measures (PROMs)	☐ UKKA patient experience survey<br>☐ Other patient experience survey (specify) ______<br>☐ Other patient reported outcome measure (specify) ______<br>☐ Carer experience survey (specify) ______<br>☐ Carer quality of life survey (specify) ______
No patient reported outcomes are in usual use	○

E2a. Indicate how each PROM (or other measure) reported in the previous question (E1) is used by your RSC service

Select all that apply

	Screening RSC clinic patients	Screening haemodialysis patients	Screening peritoneal dialysis patients	Screening conservatively managed patients	When symptoms/issues suspected	Other use
IPOS-Renal	☐	☐	☐	☐	☐	☐
POS-S Renal	☐	☐	☐	☐	☐	☐
ESASr-Renal	☐	☐	☐	☐	☐	☐
EQ-5D	☐	☐	☐	☐	☐	☐
ESRD-SCLTM	☐	☐	☐	☐	☐	☐
KDQOL-36	☐	☐	☐	☐	☐	☐
KDQOL-SF	☐	☐	☐	☐	☐	☐
SF-6D	☐	☐	☐	☐	☐	☐
SF-12	☐	☐	☐	☐	☐	☐
SF-36	☐	☐	☐	☐	☐	☐
UKKA patient experience survey	☐	☐	☐	☐	☐	☐

Please specify other use for IPOS-Renal ______________________

Please specify other use for POS-S Renal ______________________

Please specify other use for ESASr-Renal ______________________

Please specify other use for EQ-5D ______________________

Please specify other use for ESRD-SCLTM ______________________

Please specify other use for KDQOL-36 ______________________

Please specify other use for KDQOL-SF ______________________

Please specify other use for SF-6D ______________________

Please specify other use for SF-12 ______________________

Please specify other use for SF-36 ______________________

Please specify other use for UKKA patient experience

survey

E2b. Indicate how the "[e1sympother]" symptom measure you have mentioned in question E1 is used by your RSC service<br><br>Select all that apply	☐ Screening RSC clinic patients<br>☐ Screening haemodialysis patients<br>☐ Screening peritoneal dialysis patients<br>☐ Screening conservatively managed patients<br>☐ When symptoms/issues suspected<br>☐ Other use
Please specify other use of the "[e1sympother]" symptom measure	________________________________
E2b. Indicate how the "[e1qolother]" quality of life measure you have mentioned in question E1 is used by your RSC service<br><br>Select all that apply	☐ Screening RSC clinic patients<br>☐ Screening haemodialysis patients<br>☐ Screening peritoneal dialysis patients<br>☐ Screening conservatively managed patients<br>☐ When symptoms/issues suspected<br>☐ Other use
Please specify other use of the "[e1qolother]" quality of life measure	________________________________
E2b. Indicate how the "[e1otherotherprem]" patient experience survey you have mentioned in question E1 is used by your RSC service<br><br>Select all that apply	☐ Screening RSC clinic patients<br>☐ Screening haemodialysis patients<br>☐ Screening peritoneal dialysis patients<br>☐ Screening conservatively managed patients<br>☐ When symptoms/issues suspected<br>☐ Other use
Please specify other use of the "[e1otherotherprem]" patient experience survey	________________________________
E2b. Indicate how the "[e1otherotherprom]" patient reported outcome measure you have mentioned in question E1 is used by your RSC service<br><br>Select all that apply	☐ Screening RSC clinic patients<br>☐ Screening haemodialysis patients<br>☐ Screening peritoneal dialysis patients<br>☐ Screening conservatively managed patients<br>☐ When symptoms/issues suspected<br>☐ Other use
Please specify other use of the "[e1otherotherprom]" patient reported outcome measure	________________________________
E2b. Indicate how the "[e1othercarerprem]" carer experience survey you have mentioned in question E1 is used by your RSC service<br><br>Select all that apply	☐ Screening RSC clinic patients<br>☐ Screening haemodialysis patients<br>☐ Screening peritoneal dialysis patients<br>☐ Screening conservatively managed patients<br>☐ Used where symptoms/issues suspected<br>☐ Other use
Please specify other use of the "[e1othercarerprem]" carer experience survey	________________________________
E2b. Indicate how the "[e1othercarerqol]" carer quality of life survey you have mentioned in question E1 is used by your RSC service<br><br>Select all that apply	☐ Screening RSC clinic patients<br>☐ Screening haemodialysis patients<br>☐ Screening peritoneal dialysis patients<br>☐ Screening conservatively managed patients<br>☐ When symptoms/issues suspected<br>☐ Other use
Please specify other use of the "[e1othercarerqol]" carer quality of life survey	________________________________

Describe the frequency of screening, who performs the

E3. Please expand on your answers to questions E1 and E2 by briefly describing your use of patient/carer-reported measures by your RSC service

If you do not use any measures, please leave this section blank

screening, and any other information you think relevant to understand your use of these measures ________________________

E4a. Which of the following nutrition and function or frailty assessment tools are routinely used by your RSC service?

Select all that apply

Nutritional assessment tools	☐ Subjective Global Assessment (SGA)<br>☐ Short Patient Generated-SGA (PG-SGA)<br>☐ Full PG-SGA<br>☐ Other ______
Function or frailty assessment tools	☐ Karnofsky / AKPS<br>☐ Clinical (Rockwood) Frailty Scale<br>☐ Other _____
No tools assessing nutrition, function, or frailty are in routine use	○

How does your service use the nutritional and frailty screening tools indicated above?

	Screening RSC clinic patients	Screening haemodialysis patients	Screening peritoneal dialysis patients	Screening conservatively managed patients	When symptoms/issues suspected	Other use
SGA	☐	☐	☐	☐	☐	☐
Short patient generated SGA	☐	☐	☐	☐	☐	☐
Full PG-SGA	☐	☐	☐	☐	☐	☐

Please specify other SGA use	________________________
Please specify other short patient generated SGA use	________________________
Please specify other full PG-SGA use	________________________
How does your service use the "[e4nutother]" nutritional assessment you mentioned above?	☐ Screening RSC clinic patients<br>☐ Screening haemodialysis patients<br>☐ Screening peritoneal dialysis patients<br>☐ Screening conservatively managed patients<br>☐ When symptoms/issues suspected<br>☐ Other use

Please specify other use of "[e4nutother]" nutritional assessment

____

How does your service use the function or frailty screening tools indicated above?	Screening RSC clinic patients	Screening haemodialysis patients	Screening peritoneal dialysis patients	Screening conservatively managed patients	When symptoms/issues suspected	Other use
Karnofsky / AKPS	☐	☐	☐	☐	☐	☐
Clinical (Rockwood) Frailty Scale	☐	☐	☐	☐	☐	☐

Please specify other use for Karnofsky / AKPS ________________________________

Please specify other use for Clinical (Rockwood) Frailty Scale ________________________________

How does your service use the "[e4funcother]" function or frailty assessment measure?

- ☐ Screening RSC clinic patients
- ☐ Screening haemodialysis patients
- ☐ Screening peritoneal dialysis patients
- ☐ Screening conservatively managed patients
- ☐ When symptoms/issues suspected
- ☐ Other use

Please specify other use for "[e4funcother]" assessment ________________________________

E5. Please expand on your answers to Question E4 by briefly describing your use of nutrition, function, and/or frailty assessments by your RSC service

Describe the frequency of screening, who performs the screening, and any other information you think relevant to understand your use of these measures ____

E6. Are there any other screening programs or routine patient assessments that have not been covered in the previous questions?

Please describe any other aspects of your RSC service that you think relevant ____

To mark this section as complete and return to the list of Sections, please click "Submit"

If you have not completed this section, please click "Save and Return Later" to save your answers, then when you return to the survey you can click "Survey Queue" in the top right corner

Once you have completed all sections, click the button to the left "Begin Survey" to finalise and submit the survey

Please indicate which staff members have contibuted to the completion of this survey

Multiple people can contribute to completing this survey. As many roles as apply can be indicated.

Please indicate your role at the unit

Multiple people can contribute to completing this survey. As many roles as apply can be indicated.

☐ Allied health professional (specify) ______
☐ IT/Data manager
☐ Nephrologist
☐ Nurse
☐ Palliative Care Physician
☐ Research coordinator/Trials nurse
☐ Other (specify) ______

Thank you for taking the time to contribute to this project. Your responses will be very helpful in informing future work to support and develop Renal Supportive Care research and service provision

If you believe that you have not completed all the sections, you can return to other sections by clicking "Survey Queue" now in the top right corner.

Would you like a copy of the data entered sent to your unit?	◯ Yes<br>◯ No<br>(Responses will be emailed to the primary contact for the unit (the individual who first received the survey invitation))

Once you have completed all sections, click the button to the left "Begin Survey" to finalise and submit the survey

Please indicate which staff members have contributed to the completion of this survey

Multiple people can contribute to completing this survey. As many roles as apply can be indicated.

Please indicate your role at the unit

Multiple people can contribute to completing this survey. As many roles as apply can be indicated.

☐ Allied health professional (specify) ______
☐ IT/Data manager
☐ Nephrologist
☐ Nurse
☐ Palliative Care Physician
☐ Research coordinator/Trials nurse
☐ Other (specify) ______

Thank you for taking the time to contribute to this project.

Your responses indicate that you do not have a dedicated renal supportive care service, so no further answers are required. The information you have already provided will be very helpful in informing future work to support and develop Renal Supportive Care research and service provision.

Please use this final space below to provide us with any additional comments on RSC services, whether regarding your unit in particular or regarding RSC in general.

If you believe you have made a mistake, you can return to the list of Sections by clicking the "Survey Queue" button in the top right corner.

If you have no further comments just click "Submit" and the survey will be complete.

Additional Comments

Would you like a copy of the data entered sent to your unit? (Data will be sent to the primary contact email (the individual who received the survey invitation))

○ Yes
○ No

Checklist for Reporting Of Survey Studies (CROSS)

Section/topic	Item	Item description	Reported on page #
**Title and abstract**			
Title	1a	State the word "survey" along with a commonly used term in title or abstract to introduce the study's design.	1
**Introduction**			
Background	2	Provide a background about the rationale of study, what has been previously done, and why this survey is needed.	1
Purpose/aim	3	Identify specific purposes, aims, goals, or objectives of the study.	1
**Methods**			
Study design	4	Specify the study design in the methods section with a commonly used term (e.g., cross-sectional or longitudinal).	1,2
Data collection methods	5a	Describe the questionnaire (e.g., number of sections, number of questions, number and names of instruments used).	2, supplementary methods
	5b	Describe all questionnaire instruments that were used in the survey to measure particular concepts. Report target population, reported validity and reliability information, scoring/classification procedure, and reference links (if any).	2, supplementary methods
	5c	Provide information on pretesting of the questionnaire, if performed (in the article or in an online supplement). Report the method of pretesting, number of times questionnaire was pre-tested, number and demographics of participants used for pretesting, and the level of similarity of demographics between pre-testing participants and sample population.	2, supplementary methods
	5d	Questionnaire if possible, should be fully provided (in the article, or as appendices or as an online supplement).	Supplementary file
Sample characteristics	6a	Describe the study population (i.e., background, locations, eligibility criteria for participant inclusion in survey, exclusion criteria).	1,2, supplementary methods
	6b	Describe the sampling techniques used (e.g., single stage or multistage sampling, simple random sampling, stratified sampling, cluster sampling, convenience sampling). Specify the locations of sample participants whenever clustered sampling was applied.	Total population invited to participate
	6c	Provide information on sample size, along with details of sample size calculation.	

	6d	Describe how representative the sample is of the study population (or target population if possible), particularly for population-based surveys.	
Survey administration	7a	Provide information on modes of questionnaire administration, including the type and number of contacts, the location where the survey was conducted (e.g., outpatient room or by use of online tools, such as SurveyMonkey).	1, 2, supplementary methods
	7b	Provide information of survey's time frame, such as periods of recruitment, exposure, and follow-up days.	Supplementary methods
	7c	Provide information on the entry process:<br>–>For non-web-based surveys, provide approaches to minimize human error in data entry.<br>–>For web-based surveys, provide approaches to prevent "multiple participation" of participants.	2, supplementary methods
Study preparation	8	Describe any preparation process before conducting the survey (e.g., interviewers' training process, advertising the survey).	Supplementary methods
Ethical considerations	9a	Provide information on ethical approval for the survey if obtained, including informed consent, institutional review board [IRB] approval, Helsinki declaration, and good clinical practice [GCP] declaration (as appropriate).	Supplementary methods
	9b	Provide information about survey anonymity and confidentiality and describe what mechanisms were used to protect unauthorized access.	Supplementary methods
Statistical analysis	10a	Describe statistical methods and analytical approach. Report the statistical software that was used for data analysis.	2, Supplementary methods
	10b	Report any modification of variables used in the analysis, along with reference (if available).	Supplementary methods
	10c	Report details about how missing data was handled. Include rate of missing items, missing data mechanism (i.e., missing completely at random [MCAR], missing at random [MAR] or missing not at random [MNAR]) and methods used to deal with missing data (e.g., multiple imputation).	Supplementary methods
	10d	State how non-response error was addressed.	Supplementary methods
	10e	For longitudinal surveys, state how loss to follow-up was addressed.	NA
	10f	Indicate whether any methods such as weighting of items or propensity scores have been used to adjust for non-representativeness of the sample.	NA
	10g	Describe any sensitivity analysis conducted.	NA

**Results**			
Respondent characteristics	11a	Report numbers of individuals at each stage of the study. Consider using a flow diagram, if possible.	2, Table 1
	11b	Provide reasons for non-participation at each stage, if possible.	
	11c	Report response rate, present the definition of response rate or the formula used to calculate response rate.	2, Table 1
	11d	Provide information to define how unique visitors are determined. Report number of unique visitors along with relevant proportions (e.g., view proportion, participation proportion, completion proportion).	N/A
Descriptive results	12	Provide characteristics of study participants, as well as information on potential confounders and assessed outcomes.	2
Main findings	13a	Give unadjusted estimates and, if applicable, confounder-adjusted estimates along with 95% confidence intervals and p-values.	2, 3, Table 2
	13b	For multivariable analysis, provide information on the model building process, model fit statistics, and model assumptions (as appropriate).	N/A
	13c	Provide details about any sensitivity analysis performed. If there are considerable amount of missing data, report sensitivity analyses comparing the results of complete cases with that of the imputed dataset (if possible).	NA
**Discussion**			
Limitations	14	Discuss the limitations of the study, considering sources of potential biases and imprecisions, such as non-representativeness of sample, study design, important uncontrolled confounders.	4
Interpretations	15	Give a cautious overall interpretation of results, based on potential biases and imprecisions and suggest areas for future research.	3,4
Generalizability	16	Discuss the external validity of the results.	3, 4
**Other sections**			
Role of funding source	17	State whether any funding organization has had any roles in the survey's design, implementation, and analysis.	4
Conflict of interest	18	Declare any potential conflict of interest.	4
Acknowledgements	19	Provide names of organizations/persons that are acknowledged along with their contribution to the research.	

Supplementary Reference

S1. Ducharlet K, Brennan F, Raj R, et al. Kidney supportive care in Australia and New Zealand: an Australian and New Zealand Society of Nephrology (ANZSN) and Australian and New Zealand Society of Palliative Medicine (ANZSPM) guidance document. *Intern Med J*. 2026;56(Suppl 2):4–48. doi:10.1111/imj.70463
